# Eligibility for obesity management in Peru: Analysis of National Health Surveys from 2014 to 2022

**DOI:** 10.12688/wellcomeopenres.19498.1

**Published:** 2023-07-06

**Authors:** Antonio Bernabe-Ortiz, Rodrigo M. Carrillo-Larco

**Affiliations:** 1 Universidad Cientifica del Sur, Lima, Peru; 2 Hubert Department of Global Health, Rollins School of Public Health, Emory University, Atlanta, Georgia, USA; 3 Emory Global Diabetes Research Center of Woodruff Health Sciences Center, Emory University, Atlanta, USA

**Keywords:** adiposity, body mass index, anthropometrics, treatment, Peru

## Abstract

**Background**:

The prevalence of overweight and obesity has increased fastest in low- and middle-income countries in the last decades. Together with this rising prevalence, pharmacological and surgical interventions for obesity have emerged. How many people need these treatments is unknown. We quantified the prevalence of people in need of pharmacological and surgical treatment for obesity in Peru between 2014 and 2022.

**Methods**:

Repeated cross-sectional analysis of national health surveys in Peru was conducted. Eligibility for pharmacological treatment for obesity was: body mass index (BMI) ≥30 kg/m ^2^ or BMI ≥27 kg/m ^2^ alongside type 2 diabetes or hypertension (self-reported). Eligibility for bariatric surgery were BMI ≥40 kg/m ^2^ or BMI between 35 to 39.9 kg/m ^2^ linked to weight-related health problems. We used Poisson regressions to identify associated factors with eligibility for obesity management.

**Results**:

Across years, 260,131 people (mean age 44.0 and 54.5% were women) were studied, 66,629 (27.7%; 95% CI: 27.4% - 28.1%) were eligible for obesity medication, and 5,263 (2.5%; 95% CI: 2.4% - 2.6%) were eligible for bariatric surgery. Female sex, older age, higher socioeconomic level and study year were associated with higher probability of eligibility for both obesity medication and bariatric surgery.

**Conclusions**:

Eligibility for obesity management has increased over time in Peru. There is a need to strengthen policies to tackle overweight and obesity in Peru, acknowledging that some individuals may benefit from pharmacological and surgical interventions.

## Introduction

Although in high-income countries the rising prevalence of overweight and obesity has reached a plateau, this is not the case in low- and middle-income countries where the prevalence is still increasing ^1^. Similarly, mean body mass index (BMI) has increased, by 1 kg/m ^2^ per decade on average in Latin America ^2^, with the subsequent increasing prevalence of obesity observed over time in the region ^3^. Peru, a country in Latin America, has followed the same trend with increasing mean BMI and rising prevalence of overweight and obesity ^3, 4^.

As obesity is a major driver of the burden of chronic diseases, such as type 2 diabetes, even modest weight loss can produce health benefits ^5^. As a result, guidelines and position statements have been published to address obesity using nonpharmacological and pharmacological treatments, including metabolic surgery ^6, 7^. While global guidelines highlight that lifestyle intervention is the cornerstone for treating obesity, when these interventions fail to reach the weight loss target or did not achieve sustainable weight loss, pharmacological interventions are in order ^7, 8^, particularly for individuals with health risks ^6, 7, 9^. For example, individuals with BMI ≥27 kg/m ^2^ with at least one obesity-related comorbidity, or people with BMI ≥30 kg/m ^2^ with or without metabolic consequences, are eligible for obesity medication ^6^. Overall, even though there are pharmacological ^10^ and surgical interventions ^11^ for weight management, and there are clear guidelines, how many people meet the criteria for these interventions is unknown. This evidence is essential for health systems to understand whether they have the resources to provide pharmacological interventions for obesity for those who would most benefit from them.

Consequently, this study aimed to determine the prevalence and trends over time of eligibility for obesity medication and bariatric surgery in the general population by using nationally-representative surveys in Peru from 2014 to 2022. Additionally, we explored potential factors associated with such eligibility criteria.

## Methods

### Study design

Information from Peruvian National Demographic Surveys (ENDES in Spanish) was utilized for analyses. The ENDES is a nationally representative survey conducted yearly. Data was taken from 2014 to 2022, because since 2014, the ENDES has included a health questionnaire with information about hypertension and type 2 diabetes diagnosis. Furthermore, previous rounds of ENDES included only women.

### Population and sampling framework

The ENDES follows a bietapic sampling approach. In urban areas, the sampling units were clusters comprising block or groups of blocks with more than 2,000 individuals and an average of 140 households, whereas the secondary sampling units were the households within each of these clusters. However, in rural areas, the primary sampling units were clusters of 500 to 2,000 individuals and the secondary sampling units were the households similar to urban areas ^12^.

For this manuscript, data from participants aged ≥18 years, with complete BMI information, computed based on measured weight and height, were included. We excluded pregnant women or those who were breastfeeding at the time of the survey.

### Variables definition

Two variables were the outcomes of interest. The first one was eligibility for obesity medication ( *i.e.*, weight loss drugs), whilst the second one was eligibility for bariatric surgery. Eligibility for obesity medication were BMI ≥30 kg/m ^2^ or BMI ≥27 kg/m ^2^ with medical problems linked to obesity such as type 2 diabetes or high blood pressure ^13^. Eligibility for bariatric surgery were BMI ≥40 kg/m ^2^ or BMI between 35 to 39.9 kg/m ^2^ linked to weight-related health problems such as type 2 diabetes of high blood pressure ^14^. For both eligibility criteria, type 2 diabetes and high blood pressure levels were evaluated by self-reporting.

To describe participants and assess potential factors associated with the outcomes of interest, we also used socio-demographic and geographical variables. We included sex (female *versus* male); age (categorized as <30, 30-39, 40-49, 50-59, 60-69, and ≥70 years); education level (in years, <7, 7-11, and ≥12, compatible with primary, secondary and superior education); and socioeconomic level, computed using a wealth index based on assets and services that the participant reported having in the household following the DHS program standard methodology ^15^, and then split into quintiles. Geographic area (urban *versus* rural) was also included as well as study year (as numerical variable, but for descriptive purposes it was used as categorical).

### Statistical methods

Analyses were conducted using STATA 16 for Windows (StataCorp, College Station, TX, US). Descriptive statistics and estimates were calculated accounting for the complex survey design using sample strata, primary sampling units and weights, including analysis of subpopulation groups if required ^16^.

Initially, the description of variables was carried out using mean and standard deviation (SD) for numerical variables, and absolute and relative frequencies for categorical ones. Prevalence of the two outcomes of interest and their respective 95% confidence intervals (95% CI) were also estimated. Comparisons were performed using the Chi-squared test accounting for the survey design with the Rao-Scott second-order correction ^17^ for categorical variables.

Factors associated with eligibility for obesity medication and bariatric surgery were evaluated using Poisson regression models. Bivariable (crude) models were built using the outcome of interest and each of the potential associated factors, whereas multivariable models were created by including the outcome and the complete list of potential factors ( *i.e.*, exploratory analysis). Those variables with a p-value <0.05 were considered as significant.

Given the interest to assess trends over time of our outcomes of interest, a marginal model was fitted with a specific outcome and study year as the exposure of interest, adjusted for the other variables ( *i.e.*, sex, age, etc.) and then plotted and presented as figures.

### Ethics

We did not consider IRB approval mandatory as this is a secondary analysis of anonymous and freely available public data. Information do not reveal personal identifiers, and as a result, this study does not represent an ethical risk for participants. The Instituto Nacional de Estadística e Informática (INEI in Spanish), the Peruvian governmental organization responsible for ENDES data collection every year, requested informed consent from participants prior to the application of the survey.

### Data accessibility

Data used in this analysis is freely available in the webpage of the National Institute of Statistics and Informatics ( INEI).

## Results

### Description of the study population

From 2014 to 2022, out of a total of 328,167 records, 49,326 (15.0%) were excluded as subjects were aged <18 years, 4,003 (1.2%), because they were pregnant or breastfeeding women, and 14,707 (4.5%) because they did not have complete information in the variables of interest ( *i.e.*, BMI, self-report of hypertension and type 2 diabetes). Thus, data from 260,131 (79.3%) individuals were available for analysis, mean age was 44.0 (SD: 16.9) years, 54.7% were females, and 23.8% were from rural areas. Of note, during the study period, overweight ( *i.e.*, BMI ≥25 kg/m ^2^) increased from 61.2% in 2014 up to 66.8% in 2022 (p<0.001), whereas obesity ( *i.e.*, BMI ≥30 kg/m ^2^) increased from 20.9% to 27.3% in the same time period (p<0.001).

### Eligibility for obesity management

**Figure 1.**
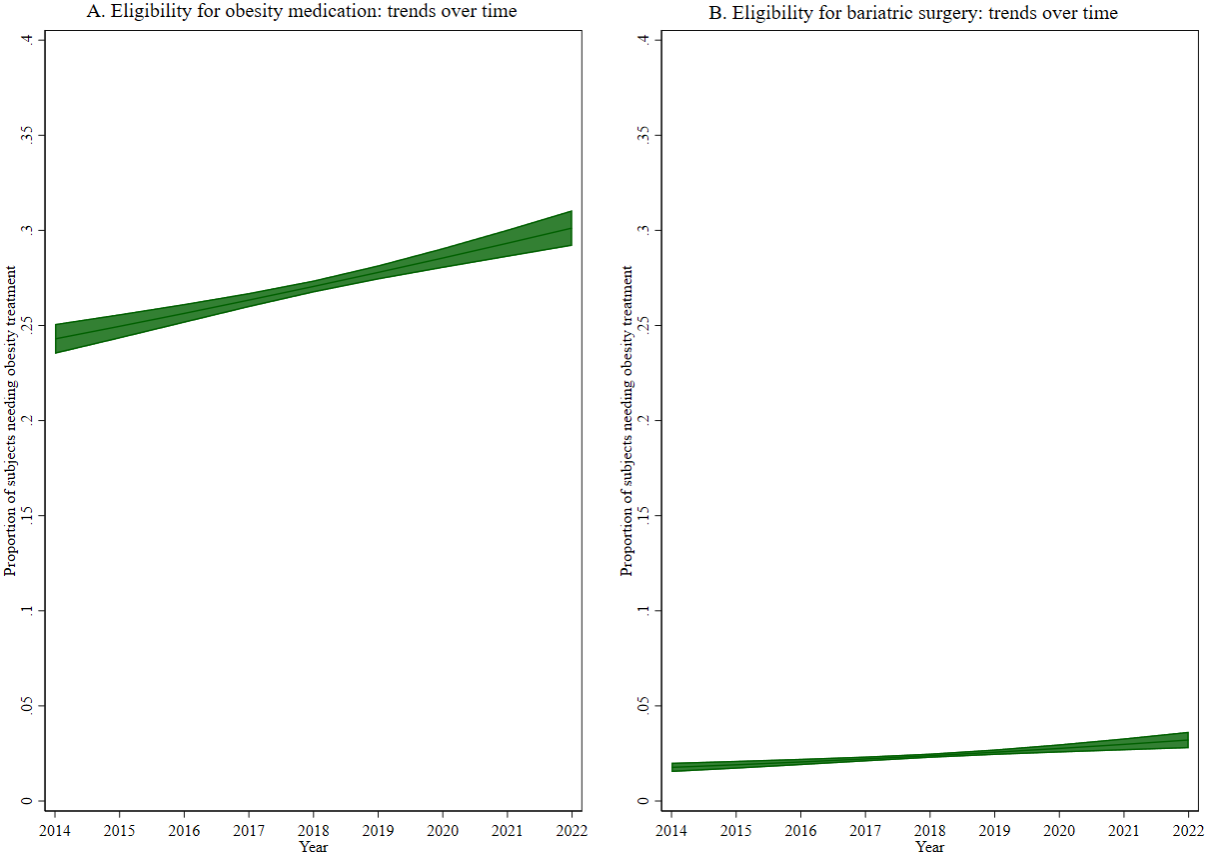
Trends over time of eligibility for ( **A**) obesity medication and ( **B**) bariatric surgery.

Over the study years and according to our definition, 66,629 (27.7%; 95% CI: 27.4% - 28.1%) subjects were eligible for obesity medication. Such eligibility was more common among females (p<0.001) and among urban dwellers (p<0.001). In addition ( Table 1), eligibility for obesity medication showed an increase with age (p<0.001), with socioeconomic level (p<0.001), and increased from 24.4% in 2014 to 30.8% in 2022 (p<0.001, see Figure 1A).

**Table 1.** Description of the study population by eligibility for obesity medication: analysis accounting for complex survey design.

	Eligibility for obesity medication		
	No (n = 193,502)	Yes (n = 66,629)	p-value *
**Sex**			<0.001
Males	90,809 (77.1%)	22,986 (22.9%)	
Females	102,693 (68.3%)	43,643 (31.7%)	
**Age (categories)**			<0.001
< 30 years	61,815 (85.7%)	11,474 (14.3%)	
30 – 39 years	53,138 (73.4%)	19,456 (26.6%)	
40 – 49 years	29,606 (66.7%)	13,943 (33.3%)	
50 – 59 years	19,591 (63.0%)	10,109 (37.0%)	
60 – 69 years	14,652 (62.9%)	7,216 (37.1%)	
70+ years	14,700 (71.8%)	4,431 (28.2%)	
**Education level**			<0.001
< 7 years	52,089 (71.9%)	17,160 (28.1%)	
7 – 11 years	77,917 (71.4%)	27,502 (28.6%)	
12+ years	54,513 (73.1%)	19,918 (26.9%)	
**Socioeconomic level**			<0.001
Very low	45,722 (84.2%)	8,459 (15.8%)	
Low	41,694 (81.7%)	9,038 (18.3%)	
Middle	35,238 (69.0%)	15,577 (31.0%)	
High	33,500 (65.9%)	16,934 (34.1%)	
Very high	37,348 (69.0%)	16,621 (31.0%)	
**Geographic area**			<0.001
Urban	117,825 (68.8%)	51,446 (31.2%)	
Rural	75,677 (83.4%)	15,183 (16.6%)	
**Study year**			<0.001
2014	19,583 (75.6%)	5,733 (24.4%)	
2015	23,753 (75.4%)	6,985 (24.6%)	
2016	22,767 (74.9%)	6,993 (25.1%)	
2017	23,254 (73.4%)	7,421 (26.6%)	
2018	23,607 (71.6%)	8,389 (28.4%)	
2019	23,003 (72.1%)	8,040 (27.9%)	
2020	14,860 (69.9%)	5,911 (30.1%)	
2021	20,833 (68.3%)	8,575 (31.7%)	
2022	21,842 (69.2%)	8,582 (30.8%)	

Proportions are weighted according to complex survey design. * P-value was estimated utilizing the Chi-squared test with the Rao-Scott second-order correction.

Eligibility for bariatric surgery was present in 5,263 (2.5%; 95% CI: 2.4% - 2.6%) and was more common among females (p<0.001) and those from urban areas (p<0.001). Eligibility increased with age (p<0.001) and with socioeconomic level (p<0.001, Table 2). Similar to eligibility for obesity medication, eligibility for bariatric surgery increased from 2.0% in 2014 to 3.3% in 2022 (p<0.001, see Figure 1B).

**Table 2.** Description of the study population by eligibility for bariatric surgery: analysis accounting for complex survey design.

	Eligibility for bariatric surgery		
	No (n = 254,868)	Yes (n = 5,263)	p-value *
**Sex**			<0.001
Males	112,508 (98.5%)	1,287 (1.5%)	
Females	142,360 (96.6%)	3,976 (3.4%)	
**Age (categories)**			<0.001
< 30 years	72,630 (99.1%)	659 (0.9%)	
30 – 39 years	71,346 (98.0%)	1,248 (2.0%)	
40 – 49 years	42,420 (96.9%)	1,129 (3.1%)	
50 – 59 years	28,673 (96.1%)	1,027 (3.9%)	
60 – 69 years	21,062 (95.7%)	806 (4.3%)	
70+ years	18,737 (97.4%)	394 (2.6%)	
**Education level**			0.14
< 7 years	67,846 (97.5%)	1,403 (2.5%)	
7 – 11 years	103,266 (97.4%)	2,153 (2.6%)	
12+ years	72,902 (97.6%)	1,529 (2.4%)	
**Socioeconomic level**			<0.001
Very low	53,692 (99.1%)	489 (0.9%)	
Low	50,263 (99.1%)	469 (0.9%)	
Middle	49,553 (97.0%)	1,262 (3.0%)	
High	48,832 (96.4%)	1,602 (3.6%)	
Very high	52,528 (97.2%)	1,441 (2.8%)	
**Geographic area**			<0.001
Urban	164,804 (97.0%)	4,467 (3.0%)	
Rural	90,064 (99.1%)	796 (0.9%)	
**Study year**			<0.001
2014	24,861 (98.0%)	455 (2.0%)	
2015	30,222 (98.1%)	516 (1.9%)	
2016	29,210 (97.8%)	550 (2.2%)	
2017	30,105 (97.7%)	570 (2.3%)	
2018	31,363 (97.6%)	633 (2.4%)	
2019	30,457 (97.7%)	586 (2.3%)	
2020	20,284 (97.2%)	487 (2.8%)	
2021	28,691 (96.6%)	717 (3.4%)	
2022	29,675 (96.7%)	749 (3.3%)	

Proportions are weighted according to complex survey design. * P-value was estimated utilizing the Chi-squared test with the Rao-Scott second-order correction.

### Factors independently associated with obesity management

In the multivariable model ( Table 3), sex, age, socioeconomic level and study year were associated with higher probability of eligibility for obesity management. Thus, compared to males, females had 36% (95% CI: 33% - 40%) and 123% (99% - 149%) more probability to be eligible for obesity medication and bariatric surgery, respectively. Age was also associated with eligibility for obesity medication and bariatric surgery, reaching the higher probability in the 60-69 group compared to those <30 years. Socioeconomic level showed a rising trend in the probability to be eligible for obesity management, reaching up to an increase of 62% (95% CI: 55% - 70%) for obesity medication and 111% (95% CI: 71% - 159%) for bariatric surgery, both in the very high socioeconomic level compared to those in the very low level. Finally, each additional year was associated with an increase of 4% (95% CI: 3% - 5%) in the eligibility for obesity medication, whereas it was associated with an increase of 8% (95% CI: 4% - 11%) in the eligibility for bariatric surgery.

**Table 3.** Factors associated with eligibility for obesity medication and bariatric surgery.

	Eligibility for obesity medication		Eligibility for bariatric surgery	
	Bivariable model	Multivariable model *	Bivariable model	Multivariable model *
	PR (95% CI)	PR (95% CI)	PR (95% CI)	PR (95% CI)
**Sex**				
Female (vs. male)	1.39 (1.36 – 1.42)	1.36 (1.33 – 1.40)	2.31 (2.07 – 2.58)	2.23 (1.99 – 2.49)
**Age (categories)**				
< 30 years	Reference	Reference	Reference	Reference
30 – 39 years	1.85 (1.78 – 1.93)	1.83 (1.76 – 1.90)	2.15 (1.80 – 2.58)	2.09 (1.74 – 2.50)
40 – 49 years	2.32 (2.23 – 2.42)	2.28 (2.19 – 2.37)	3.38 (2.85 – 4.01)	3.25 (2.74 – 3.87)
50 – 59 years	2.58 (2.48 – 2.68)	2.52 (2.42 – 2.63)	4.23 (3.56 – 5.02)	4.03 (3.37 – 4.82)
60 – 69 years	2.59 (2.48 – 2.70)	2.56 (2.44 – 2.67)	4.72 (3.95 – 5.65)	4.57 (3.79 – 5.52)
70+ years	1.97 (1.87 – 2.07)	2.10 (1.99 – 2.22)	2.81 (2.29 – 3.46)	2.83 (2.25 – 3.57)
**Education level**				
< 7 years	Reference	Reference	Reference	Reference
7 – 11 years	1.02 (0.99 – 1.05)	1.00 (0.97 – 1.03)	1.04 (0.93 – 1.17)	1.01 (0.89 – 1.14)
12+ years	0.96 (0.93 – 0.99)	0.86 (0.83 – 0.89)	0.93 (0.82 – 1.05)	0.79 (0.69 – 0.91)
**Socioeconomic level**				
Very low	Reference	Reference	Reference	Reference
Low	1.16 (1.11 – 1.21)	1.05 (0.99 – 1.11)	1.04 (0.85 – 1.27)	0.75 (0.59 – 0.97)
Middle	1.96 (1.88 – 2.05)	1.47 (1.39 – 1.55)	3.33 (2.81 – 3.94)	1.68 (1.31 – 2.14)
High	2.16 (2.07 – 2.25)	1.58 (1.49 – 1.67)	3.91 (3.33 – 4.59)	1.86 (1.46 – 2.39)
Very high	1.97 (1.89 – 2.05)	1.62 (1.55 – 1.70)	3.12 (2.66 – 3.66)	2.11 (1.71 – 2.59)
**Geographic area**				
Rural (vs. urban)	0.53 (0.52 – 0.55)	0.73 (0.70 – 0.76)	0.29 (0.26 – 0.32)	0.52 (0.44 – 0.61)
**Study year**				
Per each additional year	1.04 (1.03 – 1.05)	1.03 (1.02 – 1.04)	1.07 (1.05 – 1.09)	1.08 (1.04 – 1.11)

* Model adjusted for the listed variables (PR = prevalence ratio; 95% CI: 95% confidence intervals).

Conversely, education level and geographic area were associated with a lower probability of eligibility for obesity management. Thus, those with a higher education level ( *i.e.*, 12+ years of education) had a 14% (95% CI: 11% - 17%) lower probability of eligibility for obesity medication and 21% (95% CI: 9% - 31%) for bariatric surgery. Similarly, those in rural areas had 27% (95% CI: 24% - 30%) and 48% (95% CI: 39% - 56%) lower probabilities of being eligible for obesity medication and bariatric surgery, respectively.

## Discussion

### Main findings

The prevalence of overweight and obesity has increased in Peru, and so has the eligibility for obesity medication and bariatric surgery. According to our multivariable models, females, older subjects, and those of a higher socioeconomic level had a higher probability to be eligible for obesity medication and bariatric surgery; in contrast, those with higher education and living in rural areas showed a lower probability. Finally, our results also showed that the probability of being eligible for obesity management increased from 2014 to 2022.

### Interpretation of results

Current international obesity guidelines recommend participation in high-intensity programs ( *i.e.*, 14 or more counselling sessions) for at least six months. After that, preventing weight regain can be achieved by participating in a one-year weight-loss maintenance program with at least monthly counselling ^18^. Weight loss medications in addition to behavioral-based strategies increase weight loss and reduce the risk of developing co-morbid conditions ( *i.e.*, type 2 diabetes); however, the use of such drugs have been associated with higher rates of side effects ^19^.

The evidence herein provided is essential for Peruvian health system, and perhaps other health systems in Latin America, to understand the potential needs to provide pharmacological and surgical interventions for obesity. This is relevant because according to a previous cohort study in eight large healthcare organizations in the US, weight-loss medications are rarely prescribed (1.3% of the total cohort) to eligible patients ^20^. In participants with overweight or obesity, 2.4 mg of semaglutide once weekly plus lifestyle intervention was associated with sustained, clinically relevant reduction in body weight ^21^. Nevertheless, according to this last study, nausea and diarrhea were the most common adverse events with semaglutide, but they were typically transient and mild-to-moderate in severity.

Regarding bariatric surgery, despite the increasing rates of obesity in the US and the improved surgery techniques over the last quarter-century, the number of surgeries has only marginally increased from 1993 to 2016 ^22^. Moreover, a more recent paper in the same setting estimated that, despite the health benefits of bariatric surgery ( *i.e.*, long-term all-cause mortality, life expectancy, incidence of obesity-related conditions) ^23, 24^, only 1% of eligible patients for metabolic surgery were treated appropriately in 2018 ^25^. These results suggest that broader access to bariatric surgery for eligible people may reduce the long-term sequelae and provide population-level benefits derived from weight loss in high-risk populations. Regardless of pharmacological or surgical treatment, we would expect the rates to be much lower in Peru (in comparison to the figures presented for the US) ^20, 25^. Thus, the gap to provide people with pharmacological treatment for obesity in Peru is expected to be much wider than it is for other noncommunicable diseases ( *e.g.*, hypertension) ^26^.

### Public health relevance

Peru has a fragmented healthcare system. Overall, the public sector is dependent on the Ministry of Health, whereas the social security system depends on the Ministry of Labor and Employment Promotion ^27^. In December 2020, a document was published to provide evidence-based clinical recommendations for surgical management of obesity among adults ^28^ for those with social insurance. Nevertheless, no document available exists about the use of obesity medication. On the other hand, the Peruvian Ministry of Health (public sector) approved the National Plan to Prevent and Control Overweight and Obesity taking advantage of the COVID-19 context in March 2022 ^29^. The document focuses on the articulation of strategic interventions to address overweight and obesity, the promotion of interventions for healthy nutrition and physical activity in diverse environments (household, school, university, among others), the increase of coverage and access to healthcare services for individuals with overweight and obesity; and the development of education strategies to promote healthy lifestyles (virtually using mHealth) ^19^ as well as mechanisms of follow-up. Despite this, specific and individualized strategies to tackle the problem of obesity have not been proposed. Thus, our results fulfill an information gap about the potential need of a more specific obesity management in our population considering both nonpharmacological and pharmacological interventions following strong evidence-base guidelines.

### Strengths and limitations

This analysis benefits from utilizing national representative health surveys in Peru. In addition, short-term trends were assessed using data from different continuous years, from 2014 to 2022. However, this study has limitations that deserve discussion. First, causality cannot be established given the cross-sectional nature of the surveys. Second, self-report conditions, mainly hypertension and type 2 diabetes, were used for eligibility calculations. Thus, eligibility may be underestimated as usually individuals are not aware of having chronic conditions. As a result, our findings can be conservative regarding the need of obesity medication and bariatric surgery. Finally, only some sociodemographic and geographical variables were used for describing potential factors associated with eligibility for obesity management. Nevertheless, we still deliver reliable and actionable prevalence estimates, as well as a preliminary characterization of the population who would most likely benefit from pharmacological and surgical interventions for weight loss.

## Conclusions

Eligibility for obesity pharmacological management has increased over time in Peru. Eligibility was more common among women, older age, and those in higher socioeconomic level. There is a need to strengthen policies to tackle overweight and obesity in our country, acknowledging that some individuals may benefit from pharmacological and surgical interventions.

## Data Availability

Data used in this analysis is freely available in the webpage of the National Institute of Statistics and Informatics (INEI) at https://proyectos.inei.gob.pe/microdatos/.
