# Supplementary material for: Eligibility for obesity management in Peru: Analysis of National Health Surveys from 2014 to 2022
Source: Wellcome Open Res. 2024 Aug 19;8:287. Originally published 2023 Jul 6. [Version 4] doi: 10.12688/wellcomeopenres.19498.4 (PMC11474143; doi:10.12688/wellcomeopenres.19498.4)
Supplement: Peer review history

# Reviewer report: Reviewer response for version 4

**DOI:** 10.21956/wellcomeopenres.25281.r100732

**Published:** 2024-10-23

**Authors:** Santiago Rodriguez Lopez

**Affiliations:** 1 Center for Research and Studies on Culture and Society (CIECS), CONICET Cordoba, Córdoba, Cordoba, Argentina; 2 FCEFyN, National University of Córdoba (UNC), Córdoba, Argentina

**Wellcome Open Reseach**

**Review manuscript: ‘Eligibility for obesity management in Peru: Analysis of National Health Surveys from 2014 to 2022’.**

**Comments to the authors**

 This work investigates the prevalence and trends over time in eligibility for obesity medication and bariatric surgery in the general population of Peru by using nationally-representative health surveys from 2014 to 2022. The study uses valuable secondary data and the scope is clearly presented. I believe the manuscript would benefit from addressing some issues, mainly related to the way in which results are presented, their contribution, and comparison to the existing evidence, among others.

**Specific comments:**

**Abstract**

- *Methods.* Please, provide the years of the surveys.
- *Methods.* ‘Eligibility for pharmacological treatment for obesity was: body mass index (BMI) ≥30 kg/m ^2^ or BMI ≥27 kg/m ^2^ alongside type 2 diabetes or hypertension (self-reported). Eligibility for bariatric surgery were BMI ≥40 kg/m ^2^ or BMI between 35 to 39.9 kg/m ^2^ linked to weight-related health problems’. Is not clear what those weight-related problems refer to. Since they represent type2 diabetes or hypertension (as in the case of pharmacological treatment), please, specify this.
- *Conclusions.* The authors mention that ‘some individuals’ may benefit from pharmacological and surgical interventions. Are these the ‘Female sex, older age, higher socioeconomic level and study year were associated with higher probability of eligibility for both obesity medication and bariatric surgery’ reported in the results section of the abstract? Please, specify.

**Introduction**

- The authors say that ‘lifestyle intervention’ is the cornerstone for treating obesity. Although is not the main focus of this study, I think is important to provide some reference to the macro-level determinants of obesity. Increasing evidence is reflecting the relevance of obesogenic environments that drive the increasing obesity increments in several regions of the world.

**Methods**

- ‘For this manuscript, …’. Please, refer to study/work instead of manuscript.
- ‘We excluded pregnant women or those who were breastfeeding at the time of the survey.’ Hoy many? Were other variables considered for the sample selection? I think you could provide a flowchart describing the sample selection for all surveys.
- ‘BMI ≥40 kg/m ^2^ or BMI between 35 to 39.9 kg/m ^2^ linked to weight-related health problems such as type 2 diabetes of high blood pressure ^7,16^.’ Do you mean “such as type 2 diabetes OR high blood pressure ^7,16^”?
- ‘Bivariable (crude) models were built using the outcome of interest and each of the potential associated factors,..’. I would add the word ‘alternatively’ to the end of this sentence: ‘Bivariable (crude) models were built using the outcome of interest and each of the potential associated factors, alternatively.’
- It is not clear what is the ‘marginal model’ the authors fitted to assess trends over time for the outcomes. Please, provide further details on this.

**Results**

- ‘From 2014 to 2022, out of a total of 328,167 records, 49,326 (15.0%) were excluded as subjects were aged <18 years, 4,003 (1.2%), because they were pregnant or breastfeeding women, and 14,707 (4.5%) because they did not have complete information in the variables of interest (i.e., BMI, self-report of hypertension and type 2 diabetes). Thus, data from 260,131 (79.3%) individuals were available for analysis’. Since you are describing the sample selection here, I would move this paragraph to the methods section (see my comment above on sample selection).
- *Table 1 and 2. *Next to the absolute number of participants in each category, please provide the corresponding %.
- *Figure 1A and B. *Legends in Y axis reads ‘Proportion of subjects needing obesity treatment’. Actually, it should refer to ‘Proportion of subjects eligible for obesity treatment’.
- *Figure 1A and B. *I find these figures somehow redundant with the information provided in tables 1 and 2; I would suggest not to include them. Otherwise, and given the differences in the proportion of subjects eligible for obesity treatment, you could easily merge both figures into one. If you decide to keep them, please provide more information on how trends over time were estimated here.
- *Eligibility for obesity management.* The authors describe how the variables are associated with the two outcomes. Given that these variables similarly behave with both obesity mediation and bariatric surgery -leading to a similar description in both paragraphs- I would merge Tables 1 and 2 into one table and group these findings in a simple paragraph. Then, you would have one table for descriptive analysis (including both outcomes) and one for association analysis.
- *Factors independently associated with obesity management.* ‘In the multivariable model (Table 3), female sex, older age, higher socioeconomic level and recentness of study year were associated with higher probability of eligibility for obesity management.’ Given that ‘obesity management’ is not an outcome per se, I would refer to both outcomes separately.
- *Factors independently associated with obesity management. *‘Finally, each additional year was associated with an increase of 4% (95% CI: 3% - 5%) in the eligibility for obesity medication, whereas it was associated with an increase of 8% (95% CI: 4% - 11%) in the eligibility for bariatric surgery.’ Please, be aware that for obesity medication in the multivariate model, the increment per year is 3% not 4%.

**Discussion**

- Your results for the associations between level of education (a widely used proxy of socioeconomic position) and socioeconomic level with eligibility for obesity medication/bariatric surgery seem to go in opposite directions. Could you provide an explanation for these findings in the discussion?
- *Interpretation of results.* ‘A review using US guidelines as frameworks recommended participation in high-intensity programs (i.e., 14 or more counselling sessions) for at least six months. After that, preventing weight regain can be achieved by participating in a one-year weight-loss maintenance program with at least monthly counselling ^20^.’ The authors start this subsection with this paragraph which is really difficult to follow without a previous context. Could you rephrase it or move this paragraph further in the discussion?    
- How your findings relate to those in other countries in the region? The reference for comparison seems to be the US, but what about other countries in Latin America?
- *Conclusions. ‘*Eligibility for obesity pharmacological management has increased over time in Peru.’ Do you refer to obesity medication? If so, what about bariatric surgery? In the results the authors say that there were also increments over time among the latter (although less steep).
- *Conclusions. ‘*There is a need to strengthen policies to tackle overweight and obesity in our country, acknowledging that some individuals may benefit from pharmacological and surgical interventions.’ Which individuals are the authors referring to? Please, be more specific.

Is the work clearly and accurately presented and does it cite the current literature?

Yes

If applicable, is the statistical analysis and its interpretation appropriate?

Yes

Are all the source data underlying the results available to ensure full reproducibility?

Yes

Is the study design appropriate and is the work technically sound?

Yes

Are the conclusions drawn adequately supported by the results?

Yes

Are sufficient details of methods and analysis provided to allow replication by others?

Yes

Reviewer Expertise:

Social epidemiology

I confirm that I have read this submission and believe that I have an appropriate level of expertise to confirm that it is of an acceptable scientific standard.

# Reviewer report: Reviewer response for version 4

**DOI:** 10.21956/wellcomeopenres.25281.r93996

**Published:** 2024-10-14

**Authors:** Vance Albaugh, Michael Kachmar

**Affiliations:** 1 Translational & Integrative Gastrointestinal & Endocrine Research (TIGER) Laboratory, Pennington Biomedical Research Center, Louisiana State University, 6400 Perkins Rd, Baton Rouge, LA, USA;Pennington Biomedical Research Center, Metamor Institute, Louisiana State University, Baton Rouge, LA, USA;Department of Surgery, Louisiana State University Health Sciences Center,, New Orleans,, LA, USA; 2 Surgery, Pennington Biomedical Research Foundation, Baton Rouge, Louisiana, USA

No further comments.

Is the work clearly and accurately presented and does it cite the current literature?

Partly

If applicable, is the statistical analysis and its interpretation appropriate?

I cannot comment. A qualified statistician is required.

Are all the source data underlying the results available to ensure full reproducibility?

Yes

Is the study design appropriate and is the work technically sound?

Partly

Are the conclusions drawn adequately supported by the results?

Yes

Are sufficient details of methods and analysis provided to allow replication by others?

Yes

Reviewer Expertise:

metabolic/bariatric surgery, obesity medicine

We confirm that we have read this submission and believe that we have an appropriate level of expertise to confirm that it is of an acceptable scientific standard.

# Reviewer report: Reviewer response for version 3

**DOI:** 10.21956/wellcomeopenres.24812.r85856

**Published:** 2024-08-09

**Authors:** Vance Albaugh, Michael Kachmar

**Affiliations:** 1 Translational & Integrative Gastrointestinal & Endocrine Research (TIGER) Laboratory, Pennington Biomedical Research Center, Louisiana State University, 6400 Perkins Rd, Baton Rouge, LA, USA;Pennington Biomedical Research Center, Metamor Institute, Louisiana State University, Baton Rouge, LA, USA;Department of Surgery, Louisiana State University Health Sciences Center,, New Orleans,, LA, USA; 2 Surgery, Pennington Biomedical Research Foundation, Baton Rouge, Louisiana, USA

We have reviewed the revised article and provide approval of the current draft. We do believe the article would be further enhanced if the following points were addressed but it is acceptable in its current format:

- **Figure 1 Legend and Readability:** The legend for Figure 1 should be expanded to provide sufficient information so the figure is understandable on its own. Additionally, the y-axis font size should be increased for better readability.
- We appreciate the authors for their attention to detail and thorough revisions. We believe this has improved the clarity and comprehensiveness of their manuscript and hope they do too.

Is the work clearly and accurately presented and does it cite the current literature?

Partly

If applicable, is the statistical analysis and its interpretation appropriate?

I cannot comment. A qualified statistician is required.

Are all the source data underlying the results available to ensure full reproducibility?

Yes

Is the study design appropriate and is the work technically sound?

Partly

Are the conclusions drawn adequately supported by the results?

Yes

Are sufficient details of methods and analysis provided to allow replication by others?

Yes

Reviewer Expertise:

metabolic/bariatric surgery

We confirm that we have read this submission and believe that we have an appropriate level of expertise to confirm that it is of an acceptable scientific standard, however we have significant reservations, as outlined above.

# Response

**Published:** 2024-08-09

**Authors:** Antonio Bernabe-Ortiz

**Affiliations:** Universidad Cientifica del Sur, Peru

**Comment: Figure 1 Legend and Readability:** The legend for Figure 1 should be expanded to provide sufficient information so the figure is understandable on its own. Additionally, the y-axis font size should be increased for better readability.

***Response:*** Thanks for the comment. We have resized the Figure 1 to increase font size of y-axis. In addition, we have rephrased the legend of that figure. Now it reads: "Trends over time of the proportion of subjects with eligibility for ( **A**) obesity medication and ( **B**) bariatric surgery (2014-2022)."

# Reviewer report: Reviewer response for version 3

**DOI:** 10.21956/wellcomeopenres.24812.r85857

**Published:** 2024-06-10

**Authors:** Mary O'Kane

**Affiliations:** 1 Leeds Teaching Hospitals NHS Trust, Leeds, UK

I managed to read the revised version and response to comments. I am happy to approve.

Is the work clearly and accurately presented and does it cite the current literature?

Yes

If applicable, is the statistical analysis and its interpretation appropriate?

I cannot comment. A qualified statistician is required.

Are all the source data underlying the results available to ensure full reproducibility?

Yes

Is the study design appropriate and is the work technically sound?

Yes

Are the conclusions drawn adequately supported by the results?

Yes

Are sufficient details of methods and analysis provided to allow replication by others?

Yes

Reviewer Expertise:

Nutrition, dietetics, obesity, bariatric surgery, guidelines

I confirm that I have read this submission and believe that I have an appropriate level of expertise to confirm that it is of an acceptable scientific standard.

# Reviewer report: Reviewer response for version 2

**DOI:** 10.21956/wellcomeopenres.22020.r71898

**Published:** 2024-05-11

**Authors:** Vance Albaugh, Michael Kachmar

**Affiliations:** 1 Translational & Integrative Gastrointestinal & Endocrine Research (TIGER) Laboratory, Pennington Biomedical Research Center, Louisiana State University, 6400 Perkins Rd, Baton Rouge, LA, USA;Pennington Biomedical Research Center, Metamor Institute, Louisiana State University, Baton Rouge, LA, USA;Department of Surgery, Louisiana State University Health Sciences Center,, New Orleans,, LA, USA; 2 Surgery, Pennington Biomedical Research Foundation, Baton Rouge, Louisiana, USA

This is a peer review of an article for the journal Wellcome Open Research entitled, "Eligibility for Obesity Management in Peru: Analysis of National Health Surveys from 2014 to 2022." This is a longitudinal analysis of public health surveys that were administered as part of the Peruvian national demographic survey from the years 2014 to 2022. Prior years were not included because they only included men. The surveys are all self-reported data, and only hypertension and diabetes are collected in terms of chronic diseases. The purpose of the manuscript was to determine the percentage of the population or trends over time of individuals being eligible for either metabolic/bariatric surgery or anti-obesity medications. The eligibility criteria for these that is defined in the manuscript are older criteria, which are no longer used by most professional societies. However, over time the authors note that the prevalence of obesity is worsening in Peru, and as expected the eligibility for bariatric surgery is also increasing. Regression analysis was used to identify variables associated with a higher probability of eligibility for both obesity medications and bariatric surgery which included female sex, older age, and higher socioeconomic level in conclusion the authors state that eligibility for obesity management has improved/increased over time. Similar to other countries, there is a need to strengthen policies to fight this health epidemic. I have a number of concerns with the article, as it is extremely limited in its analysis because of inherent limitations from the database being used. My comments are below numbered.

 1. I think it would be important for the authors to note what segment/percentage of the population the survey was completed relative to the entire population of Peru. There's over 32 million people in Peru and this survey essentially captures less than 1% of that population, which is a significant limitation. Additionally, the fact that this is all self-reported data is another limitation, especially when we know that when's people self-report their height and weight they generally overestimate their height and underestimate their body mass index/body weight. The sheer magnitude of the underestimates being provided in this manuscript should be stated and made clearer for the reader.

 2. The fact that the demographic surveys only capture self-reported high blood pressure and self-reported diabetes is a tremendous limitation to this study, again grossly underestimating the health risks of these individuals that have taken part in the survey. I think the overall trends make sense, but I have a hard time accepting any estimates from this data as not only the data but the associated diseases with obesity that would qualify individuals for surgery are not captured except for high blood pressure or diabetes. That the authors mention this in the manuscript, but this is a tremendous limitation similar to number one above.

 3. The next limitation that I think is significant is that the indications for anti-obesity medicine and bariatric surgery are not the current guidelines for obesity treatment that are endorsed internationally. This is obviously a limitation, and the years being analyzed would have these older indications, but for the purpose of this manuscript – which is to identify trends in eligibility-I would encourage the authors to use these other and more current recommendations (because we know that any numbers from the data are going to be gross underestimates to begin with).

 4. Please addend/increase the information that is in the figure legend for figure 1. Essentially it only states that what is shown is "trends over time of eligibility…" for obesity medication and bariatric surgery. Please put sufficient information in the graphic so that it is understandable completely on its own. These graphs are difficult to interpret. The y-axis font is very small and hard to read.

 5. While we appreciate the sentiment of this article is to underscore the growing obesity crisis in Latin America, and more specifically Peru. And, we acknowledge your reference (Ng M, Fleming T, Robinson M, et al 2013) suggests a stabilization of the obesity epidemic in some developed countries. The obesity crisis certainly continues to worsen in developed countries of North America[4,5]. We are not certain your opening statement “Although in high-income countries the rise in prevalence of overweight and obesity has reached a plateau” bolsters your main argument further and given it may be factually incorrect suggest revision.

 6. Regarding your statement “As a result, guidelines and position statements have been published to address obesity using nonpharmacological and pharmacological treatments, including metabolic surgery:” Your reference regarding bariatric surgery guidelines is dated (Garvey WT, Mechanick JI, Brett EM, et al. 2013). This article provides a comprehensive review and grading of a decades old evidence base for recommendations in the management of obesity. Regarding surgery, it provides evidence both in line with historically accepted guidelines (BMI >35 with obesity associated disease or BMI >40) and evidence which has been incorporated into the most recent internationally accepted guidelines (BMI >30). You base your methodology on the historic indications for surgical intervention. It may be worthwhile to acknowledge that your methods are based on the 1991 guidelines which were recently updated in 2022[2,7].

 7. In your results section it may be clearer if you delineate the directionality or the binary outcome that was associated with your findings. For example, the excerpt “In the multivariable model (Table 3), sex, age, socioeconomic level and study year were associated with higher probability of eligibility for obesity management” may be clearer to the reader if written “In the multivariable model (Table 3), female sex, older age, higher socioeconomic level and recentness of study year were associated with higher probability of eligibility for obesity management.” We would suggest similar revision throughout the manuscript for clarity.

 8. In your interpretation of results section, you state: “A review using US guidelines as frameworks recommended participation in high-intensity programs (i.e., 14 or more counselling sessions) for at least six months. After that, preventing weight regain can be achieved by participating in a one-year weight-loss maintenance program with at least monthly counselling ^18^. However, weight reduction and maintenance only using lifestyle changes alone are difficult.” We believe you are trying to set up an argument that intensive lifestyle and behavioral modification is a difficult treatment strategy regarding adherence with only modest and variable long-term success[3]. This may be more eloquently stated for readability.

 9. Likewise, in the second paragraph of the interpretation of results, it appears you are building the argument that given the rise in prevalence of obesity in Peru, it is of the utmost importance that consideration for wider use of anti-obesity medications take place. And additionally, anti-obesity medications, such as semaglutide, provide clinically relevant reductions in body mass. However, we believe this arguments clarity is de-railed by sentence structure and the final sentence “Nevertheless, according to this last study, nausea and diarrhea were the most common adverse events with semaglutide, but they were typically transient and mild-to-moderate in severity” which does not appear to further your argument.

 10. Regarding strength and limitations, we agree with the statement “our results may be also underestimated …[regarding] eligibility recommended by international guidelines ^30,31^.” However, we believe the more important understanding is that your results not only underestimate the magnitude regarding the prevalence of obesity with indication for surgical management because of the inclusion of only a few obesity related conditions. But more importantly because the recommendation for minimum BMI has dropped.

 11. Finally, we believe your argument - the growing obesity epidemic in Peru requires recognition with pharmacologic and surgical management - would be more intensely supported by the increased magnitude of effect using the updated guidelines - as a greater number of the population is eligible for surgery. Additionally, this argument may be more simply raised examining solely the prevalence of obesity in Peru - without complicating the argument through the stratification of possible patients to pharmacologic and surgical treatment.

 12. Overall I would urge the authors to emphasize many of the limitations which are not emphasized adequately in the current manuscript. There is minimal data that is new, and most countries have increasing obesity and are experiencing similar trends to that of industrialized nations, in which obesity prevalence is increasing and obviously as obesity prevalence increases the eligibility for obesity treatments (anti-obesity medication and bariatric surgery) are also increasing in parallel. There is nothing surprising about this, but from a public health perspective it is important to make sure this is known publicly as the burden of obesity and its associated diseases are significant contributors to morbidity, mortality, and cost/expenses not only direct but also indirect expenses for national healthcare systems.

Is the work clearly and accurately presented and does it cite the current literature?

Partly

If applicable, is the statistical analysis and its interpretation appropriate?

I cannot comment. A qualified statistician is required.

Are all the source data underlying the results available to ensure full reproducibility?

Yes

Is the study design appropriate and is the work technically sound?

Partly

Are the conclusions drawn adequately supported by the results?

Yes

Are sufficient details of methods and analysis provided to allow replication by others?

Yes

Reviewer Expertise:

metabolic/bariatric surgery

We confirm that we have read this submission and believe that we have an appropriate level of expertise to state that we do not consider it to be of an acceptable scientific standard, for reasons outlined above.

## References

[1] : Unmet health needs of uninsured adults in the United States. *JAMA* .2000;284(16) : 10.1001/jama.284.16.2061 2061-9 10.1001/jama.284.16.2061 11042754

[2] : 2022 American Society of Metabolic and Bariatric Surgery (ASMBS) and International Federation for the Surgery of Obesity and Metabolic Disorders (IFSO) Indications for Metabolic and Bariatric Surgery. *Obes Surg* .2023;33(1) : 10.1007/s11695-022-06332-1 3-14 10.1007/s11695-022-06332-1 36336720 PMC9834364

[3] : Maintenance of Lost Weight and Long-Term Management of Obesity. *Med Clin North Am* .2018;102(1) : 10.1016/j.mcna.2017.08.012 183-197 10.1016/j.mcna.2017.08.012 29156185 PMC5764193

[4] : US Obesity Prevalence Surged Over the Past Decade. *JAMA* .2023;330(16) : 10.1001/jama.2023.19201 1515 10.1001/jama.2023.19201 37792409

[5] : Trends in obesity across Canada from 2005 to 2018: a consecutive cross-sectional population-based study. *CMAJ Open* .2022;10(2) : 10.9778/cmajo.20210205 E439-E449 10.9778/cmajo.20210205 35609927 PMC9259440

[6] : Sociodemographic Patterns of Chronic Disease: How the Mid-South Region Compares to the Rest of the Country. *Am J Prev Med* .2017;52(1S1) : 10.1016/j.amepre.2016.09.004 S31-S39 10.1016/j.amepre.2016.09.004 27989290 PMC5171223

[7] : Gastrointestinal Surgery for Severe Obesity. *Annals of Internal Medicine* .1991;115(12) : 10.7326/0003-4819-115-12-956 956-961 10.7326/0003-4819-115-12-956 1952493

# Reviewer report: Reviewer response for version 2

**DOI:** 10.21956/wellcomeopenres.22020.r65097

**Published:** 2023-08-14

**Authors:** Mary O'Kane

**Affiliations:** 1 Leeds Teaching Hospitals NHS Trust, Leeds, UK

Thank you. My comments have been addressed.

Is the work clearly and accurately presented and does it cite the current literature?

Yes

If applicable, is the statistical analysis and its interpretation appropriate?

I cannot comment. A qualified statistician is required.

Are all the source data underlying the results available to ensure full reproducibility?

Yes

Is the study design appropriate and is the work technically sound?

Yes

Are the conclusions drawn adequately supported by the results?

Yes

Are sufficient details of methods and analysis provided to allow replication by others?

Yes

Reviewer Expertise:

Nutrition, dietetics, obesity, bariatric surgery, guidelines

I confirm that I have read this submission and believe that I have an appropriate level of expertise to confirm that it is of an acceptable scientific standard.

# Reviewer report: Reviewer response for version 1

**DOI:** 10.21956/wellcomeopenres.21599.r64129

**Published:** 2023-08-07

**Authors:** Mary O'Kane

**Affiliations:** 1 Leeds Teaching Hospitals NHS Trust, Leeds, UK

I found the article easy to read and follow. I have some comments:

**Introduction**

- In the introduction, should this sentence also include surgical interventions: “This evidence is essential for health systems to understand whether they have the resources to provide pharmacological interventions for obesity for those who would most benefit from them”?

**Variables definition**

- Was there a reason that only type 2 diabetes and hypertension, along with BMI, were chosen as the eligibility criteria for medication or bariatric surgery, given that national guidelines include a range of comorbidities? For instance: Eisenberg *et al.,* (2023) ^1^ and NICE CG189 Obesity.

**Discussion**

**Main findings**

- Reference 18 is a review paper by Wadden *et al.,* discussing the US guidelines (not an international guidelines). The paper acknowledges the difficulties in achieving weight maintenance with lifestyle changes alone, and the need for national policy initiatives addressing the environment, access to healthy food, and other areas. Hence, the need for a range of treatment options including access to medication and bariatric surgery for those with severe obesity. It may be helpful to discuss that the benefits and risks of treatment should always be considered, as the benefits may outweigh the side effects.

**Interpretation of results**

- I found interesting that those with the “higher socioeconomic level” had a higher probability for eligibility for obesity medication and bariatric surgery, as this contrasts with many developed countries in which obesity is associated with social deprivation.
- I was not sure about this statement “These results suggest that broader access to bariatric surgery for eligible people may reduce the long-term sequelae and provide population-level benefits derived from weight loss in high-risk populations”. Given the small number eligible, will bariatric surgery provide population-level benefits? Would it be more appropriate for focus on cost benefits discussed by Lester *et al.,*? 
- Given that the eligible comorbidities were limited to Type 2 diabetes and hypertension, it is likely that the numbers eligible for obesity medications and bariatric surgery is underestimated. 

Is the work clearly and accurately presented and does it cite the current literature?

Yes

If applicable, is the statistical analysis and its interpretation appropriate?

I cannot comment. A qualified statistician is required.

Are all the source data underlying the results available to ensure full reproducibility?

Yes

Is the study design appropriate and is the work technically sound?

Yes

Are the conclusions drawn adequately supported by the results?

Yes

Are sufficient details of methods and analysis provided to allow replication by others?

Yes

Reviewer Expertise:

Nutrition, dietetics, obesity, bariatric surgery, guidelines

I confirm that I have read this submission and believe that I have an appropriate level of expertise to confirm that it is of an acceptable scientific standard, however I have significant reservations, as outlined above.

## References

[1] : 2022 American Society of Metabolic and Bariatric Surgery (ASMBS) and International Federation for the Surgery of Obesity and Metabolic Disorders (IFSO) Indications for Metabolic and Bariatric Surgery. *Obes Surg* .2023;33(1) : 10.1007/s11695-022-06332-1 3-14 10.1007/s11695-022-06332-1 36336720 PMC9834364

# Response

**Published:** 2023-08-07

**Authors:** Antonio Bernabe-Ortiz

**Affiliations:** Universidad Cientifica del Sur, Peru

**REVIEWER 1** I found the article easy to read and follow. I have some comments: **Introduction** **In the introduction, should this sentence also include surgical interventions: “This evidence is essential for health systems to understand whether they have the resources to provide pharmacological interventions for obesity for those who would most benefit from them”?**

***Response:*** We have included the phrase suggested. Now it reads: “ *This evidence is essential for health systems to understand whether they have the resources to provide pharmacological or surgical interventions for obesity for those who would most benefit from them*”.  

**Variables definition** **Was there a reason that only type 2 diabetes and hypertension, along with BMI, were chosen as the eligibility criteria for medication or bariatric surgery, given that national guidelines include a range of comorbidities? For instance: Eisenberg et al., (2023)** **1** **and** **NICE CG189 Obesity** **.**

***Response:*** Thanks for pointing out this. We have clarified this in the Variables definition section by adding: “ *We only utilized these two chronic conditions as they were the only ones available in the ENDES*”. In addition, in the Strengths and limitations section, we have added: "Besides, our results may be also underestimated as the complete list of comorbities to define eligibility recommended by international guidelines (a,b) *, was not pursued*.”   References:

1. Eisenberg D, Shikora SA, Aarts E, Aminian A, Angrisani L, Cohen RV, et al. 2022 American Society for Metabolic and Bariatric Surgery (ASMBS) and International Federation for the Surgery of Obesity and Metabolic Disorders (IFSO): Indications for Metabolic and Bariatric Surgery. Surg Obes Relat Dis 2022;18(12):1345-56.
2. National Institute for Health and Care Excellence. Obesity: identification, assessment and management (CG189). Available at: www.nice.org.uk/guidance/cg189.

**Discussion: Main findings** ***Reference 18 is a review paper by Wadden et al., discussing the US guidelines (not an international guideline). The paper acknowledges the difficulties in achieving weight maintenance with lifestyle changes alone, and the need for national policy initiatives addressing the environment, access to healthy food, and other areas. Hence, the need for a range of treatment options including access to medication and bariatric surgery for those with severe obesity. It may be helpful to discuss that the benefits and risks of treatment should always be considered, as the benefits may outweigh the side effects.***

***Response:*** We have modified the paragraph according to reviewer suggestions. Now it reads: “ *A review using US guidelines as frameworks recommended participation in high-intensity programs (i.e., 14 or more counselling sessions) for at least six months. After that, preventing weight regain can be achieved by participating in a one-year weight-loss maintenance program with at least monthly counselling (a). However, weight reduction and maintenance only using lifestyle changes alone are difficult. Weight loss medications in addition to behavioral-based strategies increase weight loss and reduce the risk of developing co-morbid conditions (i.e., type 2 diabetes); however, the use of such drugs have been associated with higher rates of side effects (b). There is a need for a range of treatment options including access to medication and bariatric surgery for those with severe obesity. Discussing the benefits and risks of treatment with patients should always be considered, as the benefits must outweigh the side effects.*”   References:

1. Wadden TA, Tronieri JS, Butryn ML: Lifestyle modification approaches for the treatment of obesity in adults. Am Psychol. 2020;75(2):235–51. 32052997 10.1037/amp0000517 7027681
2. LeBlanc ES, Patnode CD, Webber EM, et al.: Behavioral and Pharmacotherapy Weight Loss Interventions to Prevent Obesity-Related Morbidity and Mortality in Adults: Updated Evidence Report and Systematic Review for the US Preventive Services Task Force. JAMA. 2018;320(11):1172–1191. 30326501 10.1001/jama.2018.7777.

 
**Interpretation of results**

- **I found interesting that those with the “higher socioeconomic level” had a higher probability for eligibility for obesity medication and bariatric surgery, as this contrasts with many developed countries in which obesity is associated with social deprivation.**

***Response:  *** In Peru, the association between socioeconomic level and obesity markers is related to the nutritional transition. Thus, Peru, is not a developed country but a middle-income country with huge inequalities. For example, a relatively recent manuscript found that education level was associated with a reduction in the prevalence of obesity, whereas socioeconomic level, assessed as wealth index, was a factor positively associated with obesity (a). So, although surprising, that is an expected result.   References:

1. Cerpa-Arana SK, Rimarachín-Palacios LM, Bernabe-Ortiz A. Association between socioeconomic level and cardiovascular risk in the Peruvian population. Rev Saude Publica 2022;56:91.

 

- **I was not sure about this statement “These results suggest that broader access to bariatric surgery for eligible people may reduce the long-term sequelae and provide population-level benefits derived from weight loss in high-risk populations”. Given the small number eligible, will bariatric surgery provide population-level benefits? Would it be more appropriate for focus on cost benefits discussed by Lester et al.? **

***Response:*** We have deleted that sentence to avoid confusion.  

- **Given that the eligible comorbidities were limited to type 2 diabetes and hypertension, it is likely that the numbers eligible for obesity medications and bariatric surgery is underestimated. **

***Response:*** We have added that as a limitation as explained in a previous comment.
